# Herpes simplex virus-1 utilizes the host actin cytoskeleton for its release from axonal growth cones

**DOI:** 10.1371/journal.ppat.1010264

**Published:** 2022-01-24

**Authors:** Kevin Danastas, Ava Larsen, Sophie Jobson, Gerry Guo, Anthony L. Cunningham, Monica Miranda-Saksena

**Affiliations:** Centre for Virus Research, The Westmead Institute for Medical Research, The University of Sydney, Westmead, Australia; University of Illinois at Chicago, UNITED STATES

## Abstract

Herpes simplex virus type 1 (HSV-1) has evolved mechanisms to exploit the host cytoskeleton during entry, replication and exit from cells. In this study, we determined the role of actin and the molecular motor proteins, myosin II and myosin V, in the transport and release of HSV-1 from axon termini, or growth cones. Using compartmentalized neuronal devices, we showed that inhibition of actin polymerization, but not actin branching, significantly reduced the release of HSV-1 from axons. Furthermore, we showed that inhibition of myosin V, but not myosin II, also significantly reduced the release of HSV-1 from axons. Using confocal and electron microscopy, we determined that viral components are transported along axons to growth cones, despite actin or myosin inhibition. Overall, our study supports the role of actin in virus release from axonal growth cones and suggests myosin V as a likely candidate involved in this process.

## Author summary

Herpes simplex virus type 1 (HSV-1) is a ubiquitous human pathogen causing cold sores and genital herpes. HSV-1 infects sensory neurons of the peripheral nervous system where it establishes a lifelong infection and cannot be cured. Reactivation is common, with the virus transported back along sensory nerves, forming new lesions, or is shed asymptomatically. Antiviral resistance is emerging to current antivirals that target viral replication, indicating the need to identify new targets for future treatment. The host cell cytoskeleton plays an important role during transport of the virus. HSV-1 is transported along axons via microtubules; however, how the virus is released from axon termini, where actin predominates, is unknown. Here we show that an intact actin cytoskeleton is required for efficient virus release from axon termini. Furthermore, we show that myosin V, an actin based molecular motor that drives transport, is essential in virus release from axon termini. Together, this study defines the mechanisms behind HSV-1 release from axon termini which will guide future directions in identifying possible therapeutic targets for HSV-1.

## Introduction

Herpes simplex virus-1 (HSV-1) is a human trophic virus capable of establishing lifelong latent infections in neurons of the peripheral nervous system of human hosts. HSV-1 infects its host via the mucosa and skin where it replicates in epithelial cells. Following this initial infection, the virus then enters and infects the sensory nerves of the dorsal root ganglia (DRG) or trigeminal ganglia neurons innervating the site of infection [1,2]. The virus is then transported via retrograde axonal transport to the neuronal cell body where the viral genome is deposited in the nucleus, establishing a lifelong latent infection [1,2]. Viral reactivation leads to virus replication in the neuronal cell body and virus transport along nerves to the nerve termini for subsequent virus spread into the skin, resulting in either recurrent disease or asymptomatic virus shedding [1,2].

*In vitro* studies have shown that HSV-1 has evolved to utilize and exploit the host neuronal cytoskeleton during virus infection and spread. HSV-1 has been shown to utilize microtubules for active long-distance travel along axons during retrograde (axon to cell body) and anterograde (cell body to axon) transport [2–8]. However, how the virus exits from axons remains unclear.

Growth cones, located at the axon termini, are highly dynamic structures that facilitate axon growth and guidance and are the site of HSV-1 transmission from axons to epithelial cells [2]. Microtubules mostly terminate at the entrance to the growth cone, and actin becomes the predominant cytoskeletal component forming protrusions along the periphery. These protrusions, also known as filopodia, are composed of long, cross-linked F-actin bundles, interspersed by a meshwork of short actin fibres, known as lamellipodia [9].

Within the growth cone, actin-associated molecular motors called myosins regulate the structure and dynamics of the actin cytoskeleton, axon growth, growth cone motility, synaptic function and mediate short-range cargo or organelle transport [10–12]. Non-muscle myosin II and myosin V are two of the most abundant myosins present in the growth cones and have been reported to play a role in HSV-1 egress from non-neuronal cells [13,14]. Non-muscle myosin II, localized in the transition zone of the growth cone, is involved in regulating actin-microtubule dynamics and limiting microtubules from entering the growth cone [12,15,16]. Non-muscle myosin V is also abundant in the growth cones and is involved in filopodia extension, and the transport of vesicles along F-actin [10,17].

Given the actin-rich composition of the growth cone, and the lack of microtubules, our aim was to determine whether actin plays a role in the final release of HSV-1 from growth cones and whether non-muscle myosin II and V are involved in this process.

We have used a compartmentalized neuronal culture system to separate axons from their cell bodies to determine how the addition of actin and myosin inhibitors alters viral release from axons. Inhibition of actin polymerization significantly decreased virus release from axons, with no effect on viral release from the cell body, or transport of viral components along axons. Conversely, inhibition of actin branching, which disrupts the lamellipodia meshwork, promoted virus release from axons. We further showed that inhibition of myosin V, but not myosin II, significantly inhibited virus release from axons. Overall, our findings show that an intact actin cytoskeleton in axonal growth cones is required for efficient virus exit from axons and suggest that following transport along microtubules, once in the growth cone, HSV-1 is transported by myosin V along the actin cytoskeleton prior to final exocytosis.

## Materials and methods

### Ethics statement

All animal research was approved by the Western Sydney Local Health District animal ethics committee (approval numbers 4256 and 4309).

### Cells and viruses

Virus stocks were passaged in Vero cells grown in Dulbecco’s modified Eagle’s medium (DMEM; Invitrogen, USA) supplemented with 9% FBS (Sigma, USA). HSV-1 GFP-US9 (Fg9) was kindly provided by Dr. Renato Brandimarti (University of Bologna, Bologna, Italy) [18].

### Antibodies

Rabbit antibody against purified HSV-1 nuclear C capsids (PTNC) (1:5500 dilution) was kindy provided by Dr. Frazer Rixon, (MRC Virology Unit, Institute of Virology, UK) [19]. Mouse monoclonal anti-non-muscle myosin IIB (1:100 dilution, ab684) was obtained from Abcam, UK. Goat polyclonal anti-myosin V (1:200 dilution, LS-C139627) was obtained from Lifespan Bioscience, USA. Rabbit polyclonal anti-kinesin family member 3A (KIF3A, 1:50 dilution, SC-50457), rabbit polyclonal anti-Rab6 (1:50 dilution, SC-310) and mouse monoclonal anti-synaptosome associated protein 25 (SNAP25, 1:20 dilution, SC-20038) were obtained from Santa Cruz, USA. Alexa Fluor 633 Phalloidin and Alexa Fluor-labelled secondary antibodies were obtained from Thermo Fisher Scientific, USA.

### Inhibitors

The following reagents and working concentrations were used for actin and myosin inhibitors: cytochalasin D (0.5 μM and 1 μM, C8273), latrunculin A (0.5 μM and 2.5 μM, L5163), CK-666 (25 μM and 50 μM, SML0006) and myoVin1 (0.5 μM and 1.5 μM, 475984), were obtain from Sigma, USA. *para*-aminoblebbistatin (5 μM and 20 μM, 22699) was purchased from Cayman Chemical, USA.

### Preparation of dissociated rat neuronal cultures

DRG neurons were obtained from 6–7-day old Wistar rat neonates as previously described [4,20,21]. Briefly, DRGs were dissociated in Hanks’ calcium- and magnesium-free solution (Sigma) plus 0.25% trypsin (Sigma) and 0.05% collagenase (Sigma) for 40 min at 37°C followed by DNase (10 mg/ml; Sigma) for 4 min at 37°C. DRGs were washed two times by centrifugation at 100 x *g* and passed through a 35% Percoll gradient (Sigma). The cell pellet was resuspended in neurobasal medium (Invitrogen) and plated for neuronal cultures in microfluidic devices or on coverslips as described below.

### Neuronal cultures in microfluidic devices

Neuronal cultures were prepared as previously described [4,21,22]. Briefly, standard neuron devices (SND450; Xona Microfluidics, USA), plasma bonded to cover glasses, were coated with 0.5 mg/ml poly-D-lysine (PDL; Sigma) in borate buffer (pH 8.5), and laminin (10 μg/ml; Sigma) at 37°C with 5% CO_2_. Rat DRG dissociated neurons were prepared as described above, and a 4 μl suspension containing approximately 40,000 neurons was added to the cell body compartment of the devices (S1 Fig). Neurons were grown in neurobasal medium supplemented with L-glutamine (4 mM; Invitrogen), B-27 supplement (2%; Life Technologies), brain-derived neurotrophic factor (BDNF; 5 ng/ml; Sigma) and 7S nerve growth factor (100 ng/ml; Sigma) for 3 days at 37°C with 5% CO_2_ to allow the axons to grow into the axonal compartment of the device prior to infection (S1 Fig).

### HSV-1 infection and treatment of neurons in microfluidic devices

Cultures were infected with HSV-1 GFP-US9 (1.7 x 10^6^ PFU in 350 μl) by addition of virus to the cell body compartment only. After 2 hours, the virus inoculum was removed, and neurons washed twice with fresh neurobasal medium. All inhibitors were added at 6 hours post-infection (hpi), with the exception of CK-666, which was added at 22 hpi. At 30 hpi, the media from both the cell body and axonal compartments was collected separately and stored at -80°C. Cultures were then fixed in 3% formaldehyde for 30 min at room temperature followed by the addition of 0.1% Triton X-100 for 4 min.

All infections were performed in conjunction with mock-infected controls where neurons in the cell body compartments were mock-infected. Control experiments were also performed to check for virus leakage between compartments during virus infection. HSV-1 was added to the cell body compartment in the absence of neurons and the medium from the axonal compartment was collected at 30 hpi. In addition, virus input controls were also conducted to assess how much input virus remained in the cell body compartment after removal of the virus inoculum. For this control, neurons in the cell body compartment were HSV-1 infected as above. The virus inoculum was then removed at 2 hpi, and the neurons were washed twice with fresh media. This was followed by the addition of fresh media which was then collected (S2 Fig). Cultures were incubated for further 28 hours (until 30 hpi) before the media was collected (S2 Fig). This control was also performed for experiments conducted with neurons cultured on coverslips. This input control showed that following removal and washing of the virus inoculum at 2 hpi, only 0.092% ± 0.01% of the inoculum was present in the media collected from the cell body compartment of devices and 0.044% ± 0.0009% was present in the media collected from neurons on coverslips.

### Neuronal cultures on coverslips

Dissociated rat DRG neurons were prepared as described above. After dissociation, 20,000 neurons were resuspended in 500 μl of neurobasal media, plated onto PDL and laminin coated plastic coverslips and cultured in neurobasal medium supplemented with L-glutamine (4 mM), B-27 supplement (2%), BDNF (5 ng/ml) and 7S nerve growth factor (100 ng/ml). Cultures were incubated for 3 days at 37°C with 5% CO_2_ to allow for axon outgrowth as previously described [20,21].

### DRG explants for transmission electron microscopy (TEM)

DRG explants were prepared from neonatal rats. DRGs were dissected, cleansed of connective tissue and placed onto PDL and laminin coated plastic coverslips and cultured in neurobasal medium supplemented with L-glutamine (4 mM), B-27 supplement (2%), BDNF (5 ng/ml) and 7S nerve growth factor (100 ng/ml) at 37°C with 5% CO^2^ for 3 days to allow for axon outgrowth as previously described [21].

### HSV-1 infection and inhibitor treatment of dissociated neuronal cultures and DRG explants for electron microscopy

DRG cultures were infected with HSV-1 GFP-US9 (0.85 x 10^6^ PFU in 500 μl for dissociated cultures or 1.7 x 10^6^ PFU in 500 μl for DRG explants). After 2 hours, the inoculum was removed, and the cultures washed two times with fresh medium. Cultures were then treated with actin or myosin inhibitors at the indicated concentrations and time points as described above. At 30 hpi, the media from dissociated cultures was collected and stored at -80°C. Dissociated cultures and DRG explants were then washed twice in phosphate buffered saline (PBS) and fixed in Karnovsky’s fixative for TEM processing [23,24]. Samples were examined using either a Phillips CM120 BioTWIN TEM at 100 kV (Phillips, Netherlands) equipped with a SIS Morada digital camera or a JEOL-1400Plus 120kV TEM at 120 kV (JEOL, Japan) equipped with a microscope integrated sCMOS camera.

### Droplet digital PCR (ddPCR)

Viral DNA from the culture media collected from the cell body and axonal compartments from microfluidic devices or dissociated neuronal cultures grown on coverslips in 24-well plates was extracted using the Isolate II Genomic DNA Kit (Bioline, UK), as per the manufacturer’s instructions. Primers for ddPCR were obtained from Sigma Aldrich, USA. Primer pairs (5’-ATCAACTTCGACTGGCCCTT-3’ and 5’-CCGTACATGTCGATGTTCAC-3’) directed against HSV-1 gD gene produced a 179 bp product [25]. Reaction volumes of 20 μl were set up containing 10 μl of 2x EvaGreen Supermix (BioRad, USA), 250 nM of both forward and reverse primers, and 5 μl of DNA sample. Droplet generation was achieved using a QX200 Droplet Generator (BioRad). Droplets were then transferred to a 96-well plate, heat-sealed, and PCR performed in a C1000 thermal cycler (BioRad). The PCR cycling conditions used were as follows: 95°C for 5 min, followed by 40 cycles of 95°C for 30 s and 60°C for 1 min, followed by a single cycle of 4°C for 5 min and 90°C for 5 min. Fluorescence of the droplets was then measured using a QX200 Droplet Reader (BioRad) and the data analysed using QuantaSoft software (BioRad). Each sample was run in duplicate with mock-infected controls and no template controls included in each run. All samples had a minimum of 10,000 accepted droplets.

ddPCR was performed due to the low level of virus released in the axonal compartment of the neuronal devices. ddPCR was also performed in conjunction with fluorescent focus assays to confirm that the presence of residual inhibitors in the culture media was not the cause of plaque reduction.

### Fluorescent focus assay (FFA)

Vero cells (80,000 cells per well) were plated in Nunc LabTek 8-well Chamber Slides (Thermo Fisher Scientific) in DMEM supplemented with 9% FBS overnight. Once confluent, 5-fold serial dilutions of each sample were prepared in DMEM supplemented with 1% FBS, and 100 μl of each serial dilution was added to each well in duplicate. After 2 hours, overlay medium (DMEM supplemented with 1% FBS and 1.6% carboxy methylcellulose) was added to the cells, and the cultures were incubated for a further 48 hours at 37°C and 5% CO_2_. Cells were washed twice in PBS and fixed for 15 min in 100% methanol. Cells were imaged on an Olympus VS120 Slidescanner microscope (Olympus, Japan) and fluorescent plaques counted using FIJI imaging software [26].

### Immunofluorescence and confocal microscopy

The cultures in microfluidic devices were immunostained *in situ* by addition of primary antibodies to both cell body and axonal compartments and incubated overnight at 4°C followed by incubation of secondary antibodies for 90 min at room temperature. The cultures were washed in PBS six times after each antibody incubation step. PBS from the last wash was then removed and replaced with Fluoromount G and neurons imaged using a Leica SP5 II confocal microscope using the Leica HyD hybrid detector (Leica Microsystems, Germany). Following acquisition, deconvolution was performed using Huygens Professional Software (SVI, The Netherlands).

### Statistical analysis

Statistical analysis was performed using GraphPad Prism 8 (GraphPad, USA) using a one-way analysis of variance (ANOVA) with Dunnett’s post-hoc analysis. Two-sided p < 0.05 was considered significant.

## Results

### Virus release from axons is significantly reduced following inhibition of actin polymerization

In this study, we used primary rat DRG neurons grown in microfluidic devices treated with inhibitors of actin polymerization to determine the role of actin in virus release from growth cones. Dissociated neurons were seeded into the cell body compartment of the devices and grown for three days to allow axons to grow through the microgrooves into the axonal compartment (S1 Fig). The cell body compartment was then infected with HSV-1 and both the cell body and axonal compartments were treated with either cytochalasin D (0.5 and 1 μM) [27] or latrunculin A (0.5 and 2.5 μM) [28] at 6 hpi. The medium from each compartment was collected at 30 hpi and processed for ddPCR and FFA.

**Fig 1:**
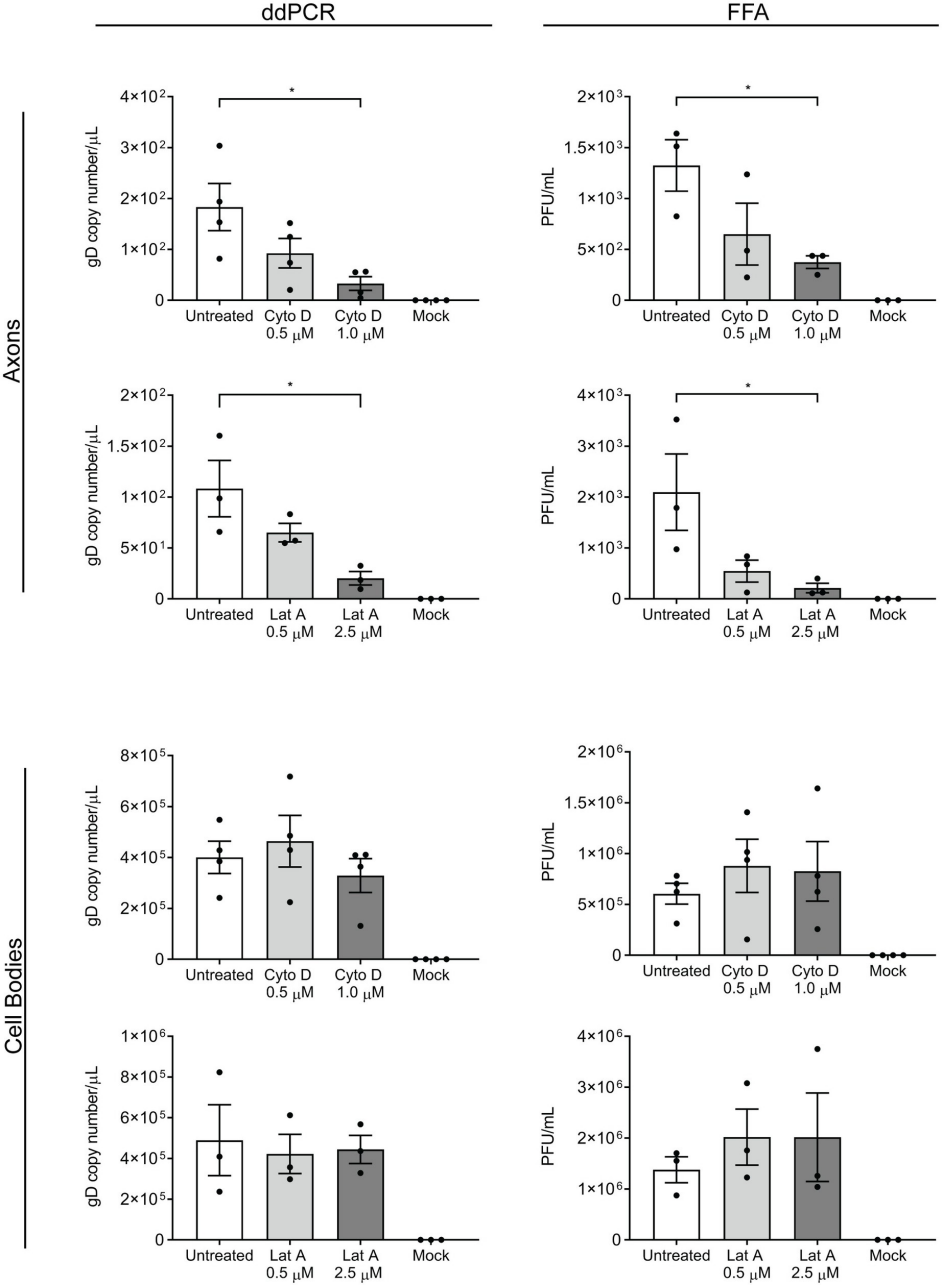
Cytochalasin D and latrunculin A inhibit the release of HSV-1 from axons. Neonatal rat DRG neurons were dissociated and seeded into the cell body compartment of microfluidic devices. Cultures were grown for 3 days to allow axons to grow into the axonal compartment before the cell body compartment was infected with HSV-1. At 6 hours post-infection (hpi), both the cell body and axonal compartments were treated with either cytochalasin D (Cyto D, 0.5 and 1 μM) or latrunculin A (Lat A, 0.5 and 2.5 μM). The medium from each compartment was collected at 30 hpi. Viral DNA was extracted from the media and analysed by droplet digital PCR (ddPCR) to measure the copy number of DNA encoding for viral envelope protein, glycoprotein D (gD). Infectious viral titres were also determined by fluorescent focus assay (FFA). Quantitation of viral release is shown for both axonal and cell body compartments following actin inhibition. Statistical analysis was performed using a one-way ANOVA with Dunnett’s multiple comparisons test (* p < 0.05). The error bars represent the standard error of the mean (SEM). n = 4 for Cyto D (n = 3 for FFA axons) and n = 3 for Lat A.

There was a marked and significant reduction in virus release into the medium in the axonal compartment following treatment with 1 μM cytochalasin D or 2.5 μM latrunculin A compared to untreated infected controls observed by both ddPCR and FFA (p < 0.05; Fig 1). No significant changes in virus release into the medium were observed in the cell body compartment following treatment with either cytochalasin D or latrunculin A compared to untreated infected controls (Fig 1).

### Virus release from axons is enhanced following inhibition of actin branching

**Fig 2:**
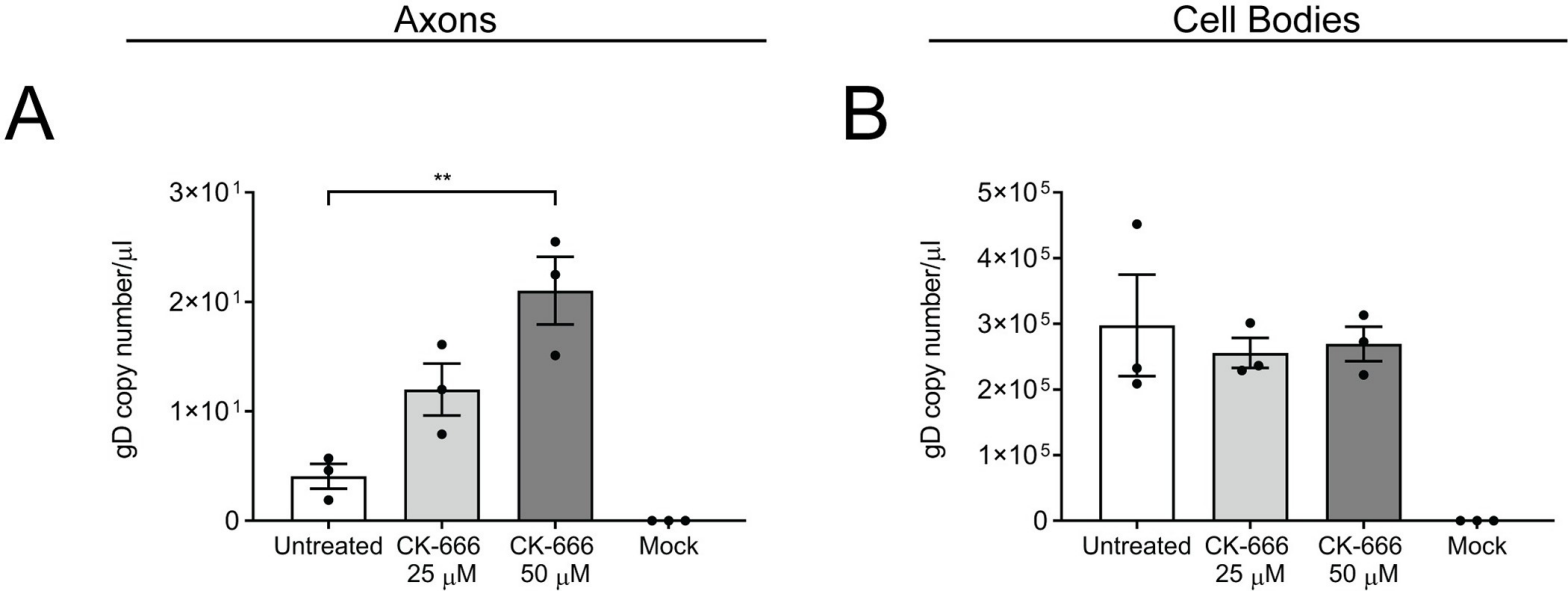
CK-666 enhances HSV-1 release from axons. Neonatal rat DRG neurons grown in microfluidic devices were infected with HSV-1 in the cell body compartment, and both compartments were treated with CK-666 (25 and 50 μM) at 22 hpi. The medium from each compartment was collected at 30 hpi. Viral DNA was extracted from the media and analysed by ddPCR to measure the copy number of DNA encoding for viral envelope protein gD. Quantitation of viral release is shown for A) axonal compartments and B) cell body compartments following actin inhibition. Statistical analysis was performed using a one-way ANOVA with Dunnett’s multiple comparisons test (** p < 0.01). The error bars represent the SEM. n = 3.

Given that inhibition of actin polymerization resulted in decreased virus release from axons, we next determined whether inhibition of actin branching would alter virus release. Rat DRGs were seeded in microfluidic devices and the cell body compartment was infected with HSV-1. At 22 hpi, both the cell body and axonal compartments were treated with the actin branching inhibitor CK-666 (25 and 50 μM) [29,30]. The medium from each compartment was then collected at 30 hpi and processed for ddPCR. Neuronal cultures were treated with CK-666 from 22–30 hpi rather than from 6–30 hpi (as with cytochalasin D and latrunculin A) to avoid detachment or clumping of the HSV-1 infected neuronal cultures, which was observed when the cultures were treated with CK-666 from 6–30 hpi. Addition of CK-666 from 22–30 hpi was found to be optimal to reliably measure and compare differences in virus release between CK-666 treated and untreated neurons (Fig 2).

There was a marked and significant increase in virus release in the axonal compartment following treatment with 50 μM CK-666 compared to the untreated infected controls observed by ddPCR (p < 0.01; Fig 2A). Due to the shorter incubation time and the low levels of virus released into the media, FFA was not sensitive enough to detect an accurate number of plaques in the axonal compartment. No significant changes in virus release were observed by ddPCR in the cell body compartment following CK-666 treatment compared to untreated infected controls (Fig 2B).

### Virus release from axons is significantly reduced following inhibition of myosin V but not myosin II

After establishing that an intact actin cytoskeleton is required for virus release from axons, we next wanted to investigate the actin-based molecular motors that may be involved in virus release from axons. Because myosin II and myosin V are predominant in growth cones, we targeted these molecular motors for inhibition studies [16,17]. As myosin II and V have been reported to play a role in HSV-1 replication and egress from non-neuronal cells [13,14], only the axonal compartment was treated with myosin II and V inhibitors. Dissociated neurons were set up in microfluidic devices as described above. The cell body compartment was infected with HSV-1 and the axonal compartment was treated with either the myosin V inhibitor myoVin1 (1.5 and 5 μM) [31] or the myosin II inhibitor, *para-*aminoblebbistatin (5 and 20 μM) [32] at 6 hpi. The medium from each compartment was collected at 30 hpi and the virus released into the media was assessed using ddPCR and FFA.

**Fig 3:**
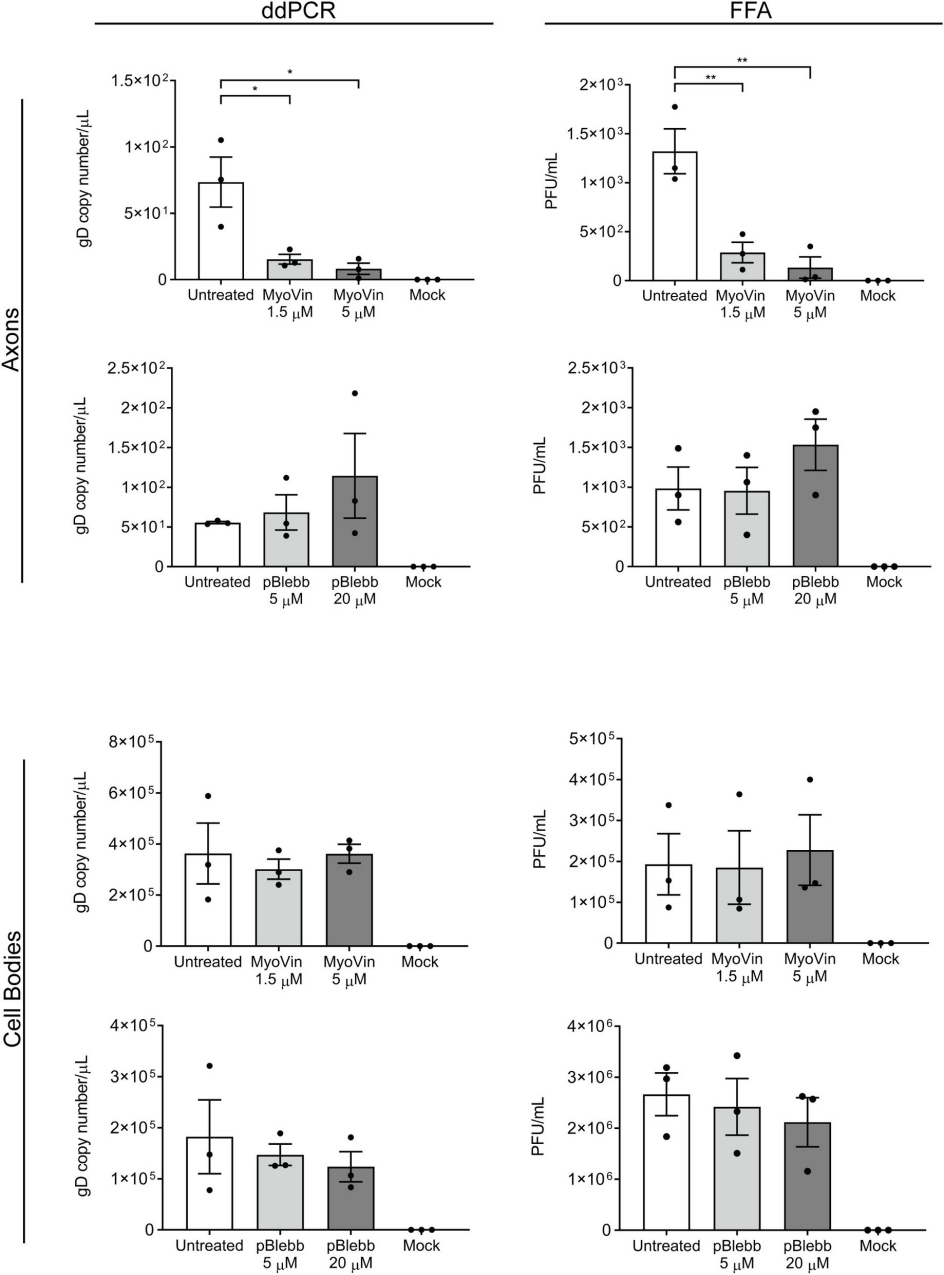
MyoVin1, but not *para*-aminoblebbistatin, inhibits HSV-1 release from axons. Neonatal rat DRG neurons grown in microfluidic devices were infected with HSV-1 in the cell body compartment, and the axonal compartments were treated with either myoVin1 (1.5 and 5 μM) or *para*-aminoblebbistatin (pBlebb, 5 and 20 μM) at 6 hpi. The medium from each compartment was collected at 30 hpi. Viral DNA was extracted from the media and analysed by ddPCR to measure the copy number of DNA encoding for viral envelope protein gD. Infectious viral titres were also determined by FFA. Quantitation of viral release into the medium is shown for axonal and cell body compartments following myosin V and II inhibition. Statistical analysis was performed using a one-way ANOVA with Dunnett’s multiple comparisons test (* p < 0.05, ** p < 0.01). The error bars represent the SEM. n = 3.

There was a marked and significant reduction in virus release in the axonal compartment following treatment of axons with myoVin1 compared to untreated infected controls observed by both ddPCR (p < 0.05) and FFA (p < 0.01) (Fig 3). Conversely, no significant change in virus release was observed in the axonal compartment following treatment of axons with *para*-aminoblebbistatin compared to untreated infected controls observed by both ddPCR and FFA (Fig 3).

No significant changes were observed in virus release in the cell body compartment following treatment of axons with either myoVin1 or *para*-aminoblebbistatin (Fig 3).

### Virus release from the neuronal cell body is not affected following myosin II or V inhibition

**Fig 4:**
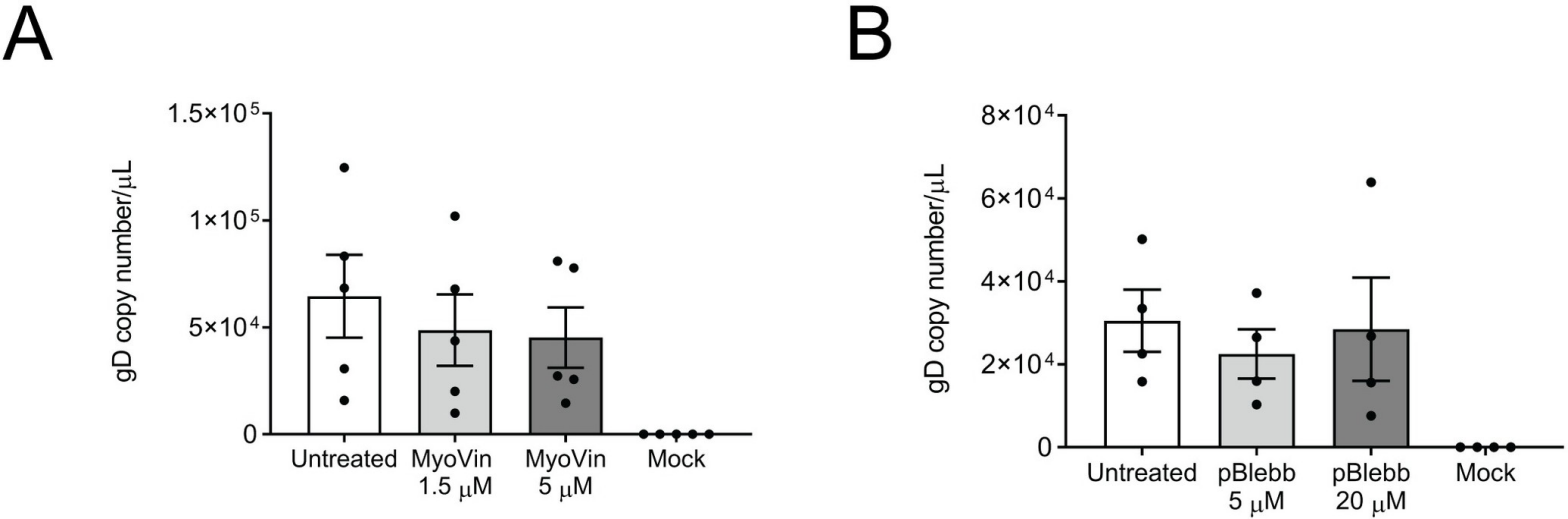
MyoVin1 and *para*-aminoblebbistatin do not affect HSV-1 release from neurons following direct treatment. Neonatal rat DRG neurons were grown on coverslips and infected with HSV-1. At 6 hpi, neurons were treated with either myoVin1 (1.5 and 5 μM) (A) or *para*-aminoblebbistatin (pBlebb, 5 and 20 μM) (B) and the media was collected at 30 hpi. Viral DNA was extracted from the media and analysed by ddPCR to measure the copy number of DNA encoding for viral envelope protein gD. The error bars represent the SEM. n = 5 for myoVin1 (A) and n = 4 for *para*-aminoblebbistatin (B).

We also investigated whether direct treatment of the neurons with either myosin II or myosin V inhibitors, rather than via the axonal compartment, had any effect on virus release. Neuronal cultures grown on coverslips were infected as above, and the neurons were directly treated at 6 hpi with either myoVin1 (1.5 μM and 5 μM) or *para*-aminoblebbistatin (5 μM and 20 μM). The media was collected at 30 hpi and processed for ddPCR. We observed no significant difference in virus release following direct treatment of the neurons with either myosin II inhibitor *para*-aminoblebbistatin or myosin V inhibitor myoVin1 compared to untreated infected controls (Fig 4).

### Actin and myosin inhibition does not affect axonal transport of HSV-1 viral proteins to growth cones

We next wanted to determine whether the replication of the virus and anterograde axonal transport of viral components was affected by actin or myosin inhibition. We also wanted to investigate whether actin or myosin inhibition affected the distribution of actin, myosin II, myosin V and other cellular proteins involved in the secretory pathways in neurons. Following collection of media, the neurons in the microfluidic devices were fixed and processed for confocal microscopy. Neurons were immunostained for viral proteins and cellular proteins including F-actin, myosin II or myosin V, and imaged using a Leica SP5 II confocal microscope.

**Fig 5:**
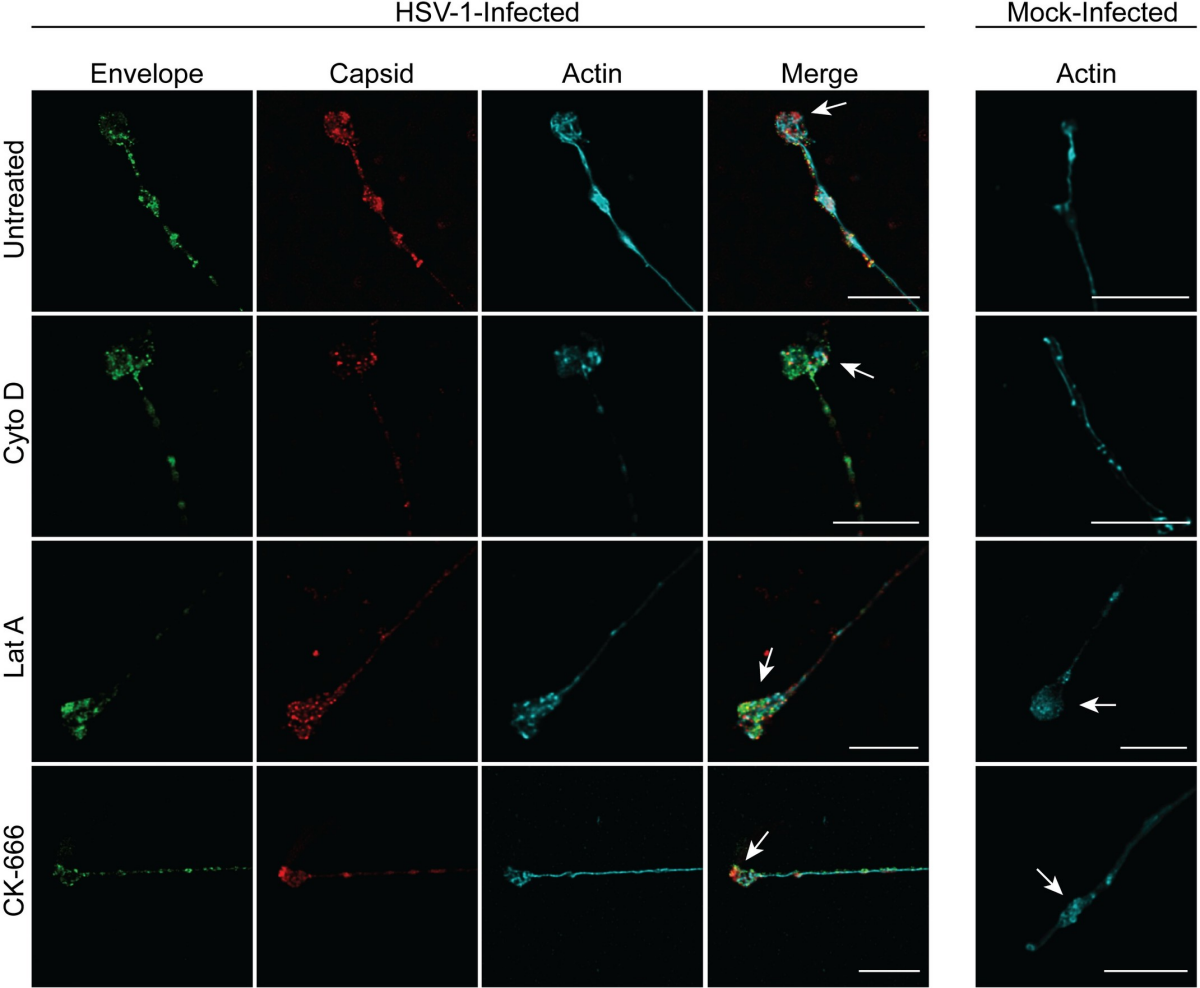
The actin inhibitors cytochalasin D, latrunculin A and CK-666, do not inhibit anterograde axonal transport of HSV-1 capsid and envelope proteins to the growth cone. Neuronal cultures in the cell body compartment were HSV-1 or mock-infected and both compartments were treated with either cytochalasin D (Cyto D) or latrunculin A (Lat A) at 6 hpi or CK-666 at 22 hpi. Neuronal cultures were fixed at 30 hpi and immunostained for HSV-1 C capsids and actin (phalloidin). Cultures were examined using a Leica SP5 II confocal microscope. Micrographs of HSV-1 infected axons showing label for viral envelope (green), viral capsid (red) and actin (cyan) following inhibition of actin by cytochalasin D, latrunculin A and CK-666. Growth cones are indicated by arrows. Scale bars = 10 μm.

**Fig 6:**
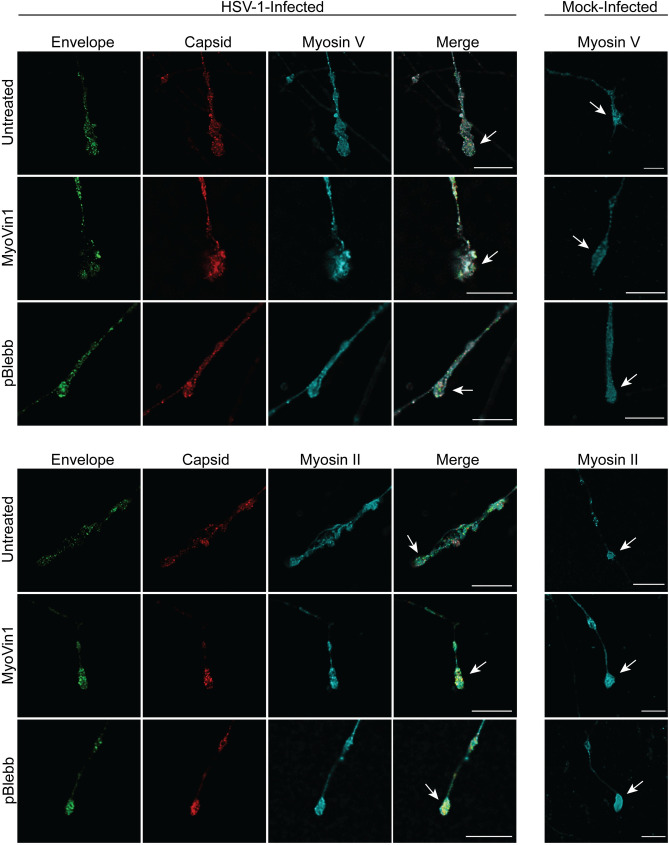
The myosin inhibitors, myoVin1 and *para*-aminoblebbistatin, do not inhibit anterograde axonal transport of HSV-1 capsid and envelope proteins to the growth cone. Neuronal cultures in the cell body compartment were HSV-1 or mock-infected and the axonal compartments were treated with either myoVin1 or *para*-aminoblebbistatin (pBlebb) at 6 hpi. Neuronal cultures were fixed at 30 hpi and immunostained for HSV-1 C capsids with either myosin V or myosin II. Cultures were examined using a Leica SP5 II confocal microscope. Micrographs of HSV-1 infected axons showing label for viral envelope (green), viral capsid (red) and either myosin V (cyan) or myosin II (cyan) following inhibition of myosin V by myoVin1 and myosin II by *para*-aminoblebbistatin. Growth cones are indicated by arrows. Scale bars = 10 μm.

Labels for viral envelope (GFP-pUS9) and capsid were present along axons, in varicosities (located at axonal branch points) and growth cones in all treatment conditions. No differences were observed in viral protein distribution along axons, axonal varicosities and growth cones following actin inhibition (Fig 5) or myosin inhibition (Fig 6) compared to untreated infected controls.

Label for actin was observed along the length of axons, particularly concentrated in growth cones and varicosities in infected and mock-infected axons. Following treatment with actin polymerization inhibitors (cytochalasin D and latrunculin A), distribution of actin became more punctate in infected axons compared to untreated infected axons (Fig 5). A similar pattern was observed in mock-infected axons treated with actin inhibitors compared to mock-infected untreated axons (Fig 5).

Label for myosin II and myosin V was present along axons, varicosities and growth cones of infected and mock-infected axons. Colocalization of myosin V with viral proteins was observed in discrete puncta in varicosities and growth cones with colocalization being more apparent in myoVin1 treated axons. No colocalization of myosin II with viral proteins was observed in either treated or untreated infected axons (Fig 6).

**Fig 7:**
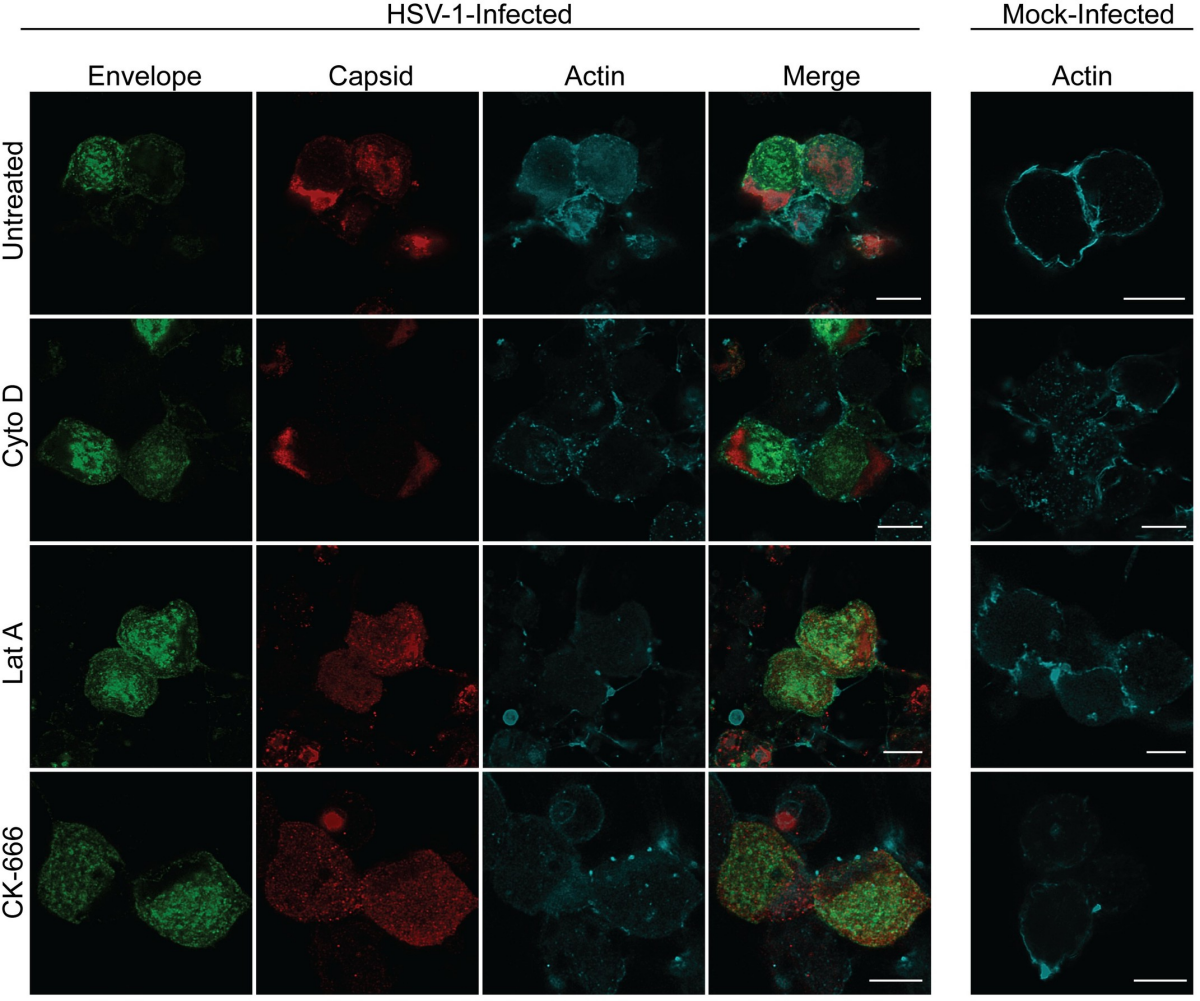
The actin inhibitors, cytochalasin D, latrunculin A and CK-666, do not affect viral replication or viral protein distribution in the cytoplasm of the cell body. Neuronal cultures in the cell body compartment were HSV-1 or mock-infected and both compartments were treated with either cytochalasin D (Cyto D) or latrunculin A (Lat A) at 6 hpi or CK-666 at 22 hpi. Neuronal cultures were fixed at 30 hpi and immunostained for HSV-1 C capsids and actin (phalloidin). Cultures were examined using a Leica SP5 II confocal microscope. Micrographs of HSV-1 infected neurons showing label for viral envelope (green), viral capsid (red) and actin (cyan) following inhibition of actin polymerization by cytochalasin D, latrunculin A and CK-666. Scale bars = 10 μm.

**Fig 8:**
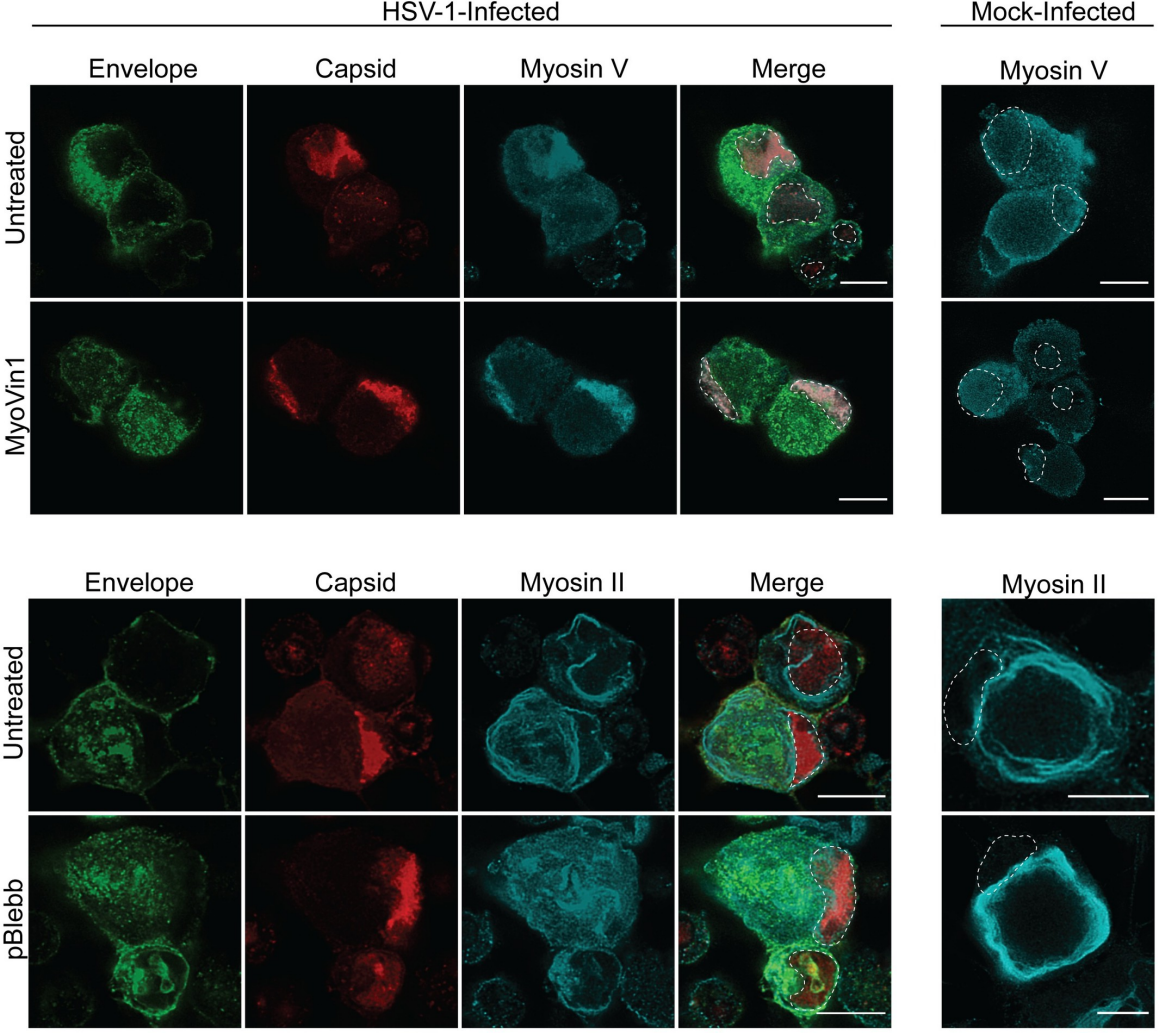
The myosin inhibitors, myoVin1 or *para*-aminoblebbistatin, do not affect viral replication or viral protein distribution in the cytoplasm of the cell body following direct treatment of the cell body compartment. Neuronal cultures were HSV-1 or mock-infected and the cell body compartment was directly treated with either myoVin1 or *para*-aminoblebbistatin (pBlebb) at 6 hpi. Neuronal cultures were fixed at 30 hpi and immunostained for HSV-1 C capsids with either myosin V or myosin II. Cultures were examined using a Leica SP5 II confocal microscope. Micrographs of HSV-1 infected neurons showing label for viral envelope (green), viral capsid (red) and myosin V (cyan) following inhibition of myosin V by myoVin1, and myosin II (cyan) following inhibition of myosin II by *para-*aminoblebbistatin. Dashed lines outline the nucleus. Scale bars = 10 μm.

Viral protein distribution in the cell body of infected neurons was unaffected by treatment with actin or myosin inhibitors (Figs 7 and 8). On the other hand, the distribution of actin changed with infection and treatment with actin inhibitors (Fig 7). A distinct actin ring was present around the periphery of the cell body of mock-infected (untreated) neurons. In infected (untreated) neurons, actin was redistributed in a diffuse pattern in the cytoplasm of the cell body. However, actin was redistributed in a punctate pattern in the cytoplasm in both mock- and HSV-1 infected neurons upon treatment with actin inhibitors (Fig 7).

Label for myosin V was observed diffusely in both the nucleus and cytoplasm of the cell body of mock-infected neurons whether untreated or whether myosin V inhibitor myoVin1 was added directly to the cell body or to the axon compartment. However, label for myosin V was concentrated mainly in the nucleus where it colocalized with the label for viral capsid in infected neurons, treated or untreated with myoVin1 (Figs 8 and S3).

Label for myosin II was mostly present in a dense ring-like pattern in the perinuclear cytoplasm and periphery of the cell body in mock-infected neurons whether untreated or whether myosin II inhibitor *para*-aminoblebbistatin was added directly to the cell body or to the axon compartment (Figs 8 and S3). On the other hand, the label for myosin II was mostly present in a striated pattern in the cytoplasm of the cell body of infected neurons treated or untreated with *para*-aminoblebbistatin (Figs 8 and S3). Label for myosin II was also detected at the nuclear membrane and weakly in the nucleus of infected neurons; however, no colocalization with the label for viral capsid was detected.

The effect of actin and myosin inhibition on the distribution of key cellular proteins involved in the secretory pathways in neurons, Synaptosomal-Associated Protein, 25kDa (SNAP-25), RAS-related protein Rab6 and kinesin KIF3A, was also investigated [33–35]. No differences were observed in the distribution of these proteins along axons, axonal varicosities and growth cones of mock- and HSV-1 infected neurons treated or untreated with either latrunculin A, CK-666 or myoVin1 (S4 and S5 Figs).

### Actin and myosin inhibition does not affect axonal transport of HSV-1 particles to varicosities and growth cones as shown by electron microscopy

Confocal microscopy showed that the anterograde transport of viral proteins along axons is unaffected by actin or myosin inhibition. Therefore, transmission electron microscopy (TEM) was used to confirm the presence of enveloped viral particles in axons, varicosities and growth cones following actin or myosin inhibition. DRG explants or dissociated neurons were grown on plastic coverslips and infected with HSV-1. Cultures were treated with inhibitors of actin (cytochalasin D and latrunculin A), myosin II (*para*-aminoblebbistatin) or myosin V (myoVin-1) at 6 hpi or with the actin branching inhibitor, CK-666, at 22 hpi. Cultures were then fixed at 30 hpi and processed for TEM.

**Fig 9:**
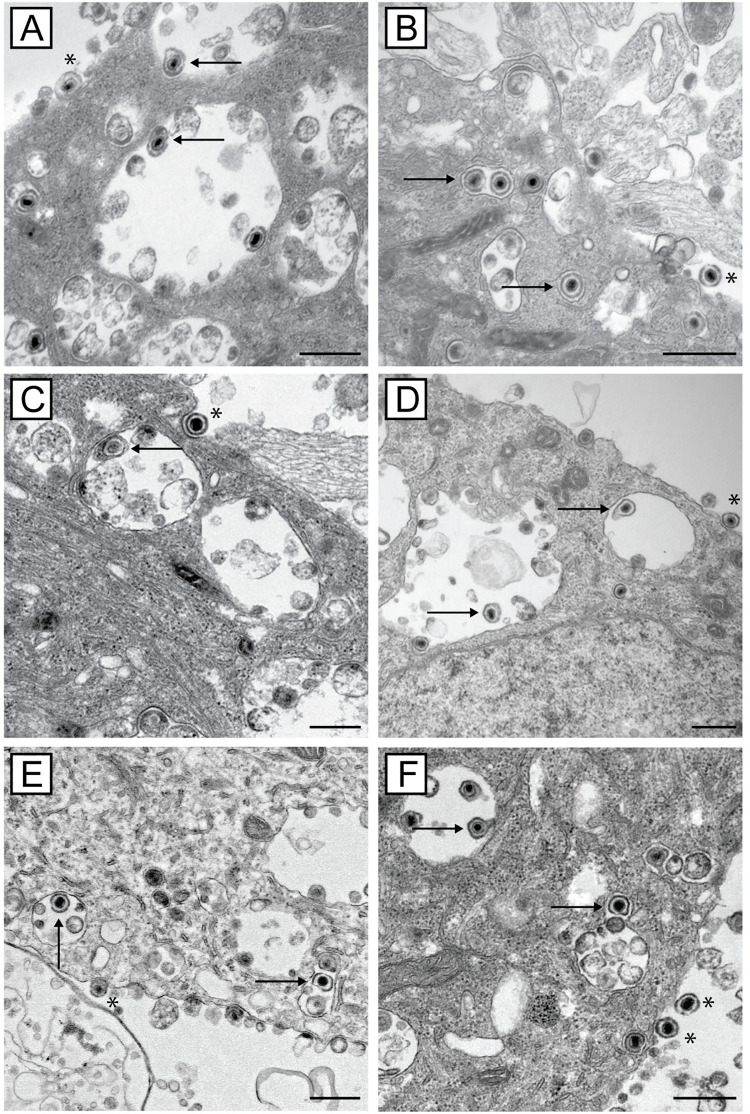
Transmission electron micrographs showing that actin and myosin inhibitors do not affect virus assembly and exit from the cell body. Neuronal cultures plated on plastic coverslips were grown for 3 days prior to HSV-1 infection. Infected cultures were either A) untreated or treated with B) cytochalasin D, C) latrunculin A, D) CK-666, E) myoVin1, F) *para*-aminoblebbistatin. Cultures were fixed at 30 hpi and examined by transmission electron microscopy. Enveloped capsids are indicated by arrows and extracellular virions are indicated by asterisks. Scale bars = 500 nm.

Numerous capsids within the nucleus, enveloped capsids (often within large vesicles) in the cytoplasm of neuronal cell bodies and extracellular virions, were present in both treated and untreated HSV-1 infected neurons (Fig 9). These observations indicate that virus replication in the nucleus, assembly in the cytoplasm and exit from the neuronal cell body were unaffected by either actin or myosin inhibition (Fig 9).

**Fig 10:**
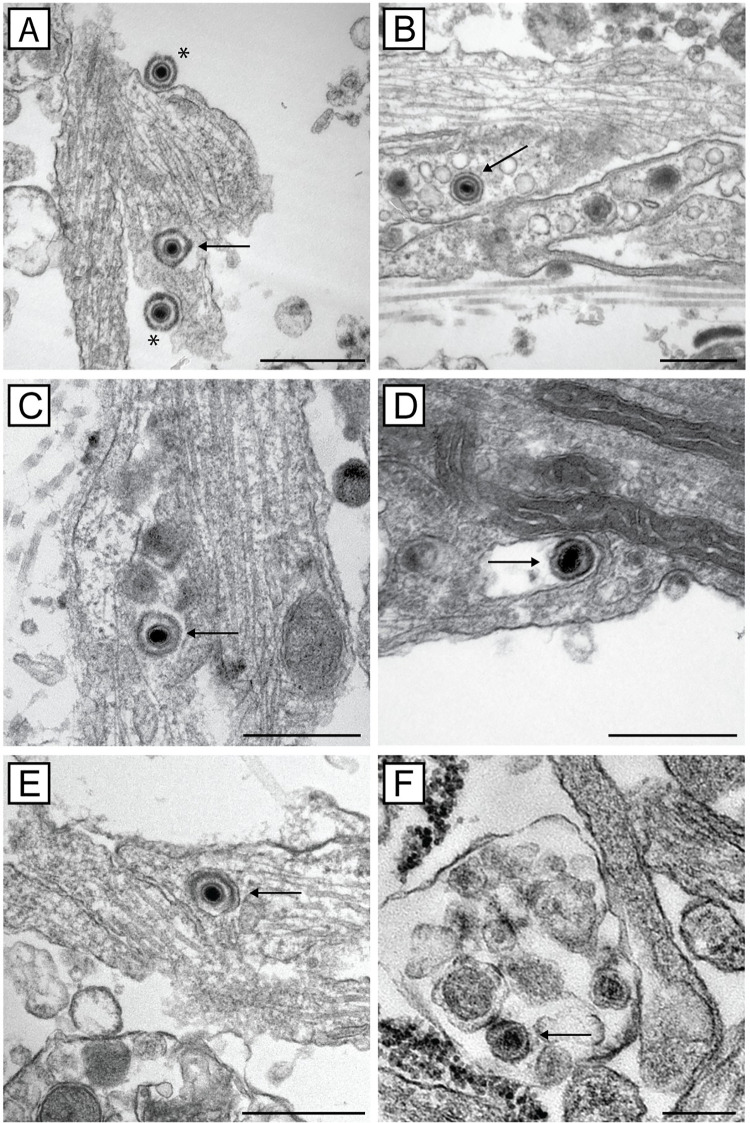
Transmission electron micrographs showing that myosin and actin inhibitors affect viral exit from axons. Neuronal cultures plated on plastic coverslips were grown for 3 days prior to HSV-1 infection. Infected cultures were either A) untreated or treated with B) cytochalasin D, C) latrunculin A, D) CK-666, E) myoVin1, F) *para*-aminoblebbistatin. Cultures were fixed at 30 hpi and examined by transmission electron microscopy. Enveloped capsids are indicated by arrows and extracellular virions are indicated by asterisks. Scale bars, = 500 nm.

Enveloped capsids (within vesicles) were also observed in varicosities and growth cones in the presence or absence of actin and myosin inhibitors (Fig 10). Extracellular viral particles were mainly observed close to axons of untreated infected neurons. These results support our confocal microscopy findings (Figs 5–8) that normal virus assembly and transport along axons to growth cones occurs in the presence of all inhibitors.

## Discussion

In this study, we used a compartmentalized neuronal culture system to separate neuronal cell bodies from axons to determine the role of actin and myosin on HSV-1 release from both the cell bodies and axons of primary sensory DRG neurons. The virus released into the culture medium of each compartment was measured using ddPCR to detect viral DNA (targeting the gene encoding for viral envelope protein gD) and FFA to detect and quantify infectious viral particles. ddPCR was performed due to the low level of virus released in the axonal compartment of the neuronal devices and to confirm that the presence of residual inhibitors in the culture media was not the cause of plaque reduction. The distribution of myosin II, myosin V, actin and viral proteins along axons and growth cones following treatment with inhibitors was visualised by confocal microscopy. In addition, the presence of viral particles in growth cones following treatment with inhibitors was visualised by TEM.

Inhibition of actin polymerization (by cytochalasin D and latrunculin A) significantly reduced virus release from axons. Conversely, inhibition of actin branching (by CK-666) significantly enhanced virus release from axons. We also observed a significant reduction in virus release from axons following inhibition of myosin V by myoVin1 but not inhibition of myosin II by *para*-aminoblebbistatin. On the other hand, inhibition of either actin polymerization, actin assembly, myosin II or myosin V had no effect on virus release from the cell body. Additionally, no treatment altered the axonal transport of viral components or viral particles along axons to growth cones as shown by confocal microscopy and TEM, respectively. Overall, our results show that an intact actin cytoskeleton is required for virus release from axons and as expected, viral transport along axons to the growth cones occurs independently of actin (but along microtubules as previously established [2]). Additionally, our data suggest that myosin V is involved in actin-based transport of HSV-1 within axonal varicosities and growth cones prior to final exocytosis.

HSV-1, like most viruses, depends on the host cytoskeleton for virus entry, replication and, as a neuroinvasive virus, has adapted mechanisms for long-distance travel along axons of sensory neurons [2,36]. Sensory neurons are highly polarized with distinct functional domains (cell body and axons) which are maintained by the coordinated regulation of the cytoskeleton (mainly microtubules and actin) dynamics [2]. In the cell body, networks of microtubules extend to the cell periphery where a thin actin cortex is present whereas, in axons, microtubules predominate in the axon shaft and mostly terminate at the entrance to the axonal growth cone at the tip of the axon with actin being the predominant cytoskeletal component of the growth cone [37]. HSV-1 has been shown to induce actin cytoskeleton rearrangement in the cytoplasm by mediating levels of cofilin-1 in neuronal and non-neuronal cell lines *in vitro* [38]. HSV-1 enhances F-actin assembly at early stages of infection to facilitate virus transport in the cytoplasm during virus entry. During virus exit, HSV-1 further alters the levels of cofilin-1 to induce actin disassembly for viral egress through the actin cortex [38]. This is in line with our observations showing that an intact actin cortex is not required for virus egress from the neuronal cell body. However, how HSV-1 interacts with the actin cytoskeleton at the axon terminus, or growth cone, is not yet known. Given the differences in the cytoskeletal structure of neuronal cell bodies and growth cones, we expect that HSV-1 utilizes different mechanisms to exit from the cell body compared to the growth cone at the axon terminus.

Several myosins, including myosin II and myosin V, are found in the actin cortex where they are involved in vesicle transport across actin and regulated exocytosis [39–41]. There is evidence that myosin II and V play a role in the transport of HSV-1 proteins and virions to the plasma membrane prior to egress from non-neuronal cells [13,14]. Inhibition of the non-muscle myosin II by the inhibitor butanedione monoxime was reported to decrease the release of extracellular HSV-1 from infected Vero or baby hamster kidney cells [14]. In addition, expression of the dominant-negative myosin Va isoform was found to reduce expression of HSV-1 glycoproteins and virus egress from infected Hela cells [13]. Our findings differ from these studies as we observed no significant differences in virus release when neurons were directly treated with inhibitors of myosin II and V. Differences in these observations could be attributed to differences in cells (neuronal vs non-neuronal cells) and type of inhibitors used. In this study, we used specific inhibitors of myosin II and V, *para*-aminoblebbistatin and myoVin1, respectively. *Para*-aminoblebbistatin binds to the myosin-ADP-P_i_ complex and interferes with phosphate release, selectively inhibiting myosin II activity [32,42], whereas myoVin1 inhibits ADP release from the actomyosin complex, selectively inhibiting myosin V activity [31]. Both myosin inhibitors act to stop the mechanism of action without altering their expression or cellular distribution [31,32,42]. As expected, we observed that the distribution of both myosin II and V was not altered following treatment of either cell body or axonal compartments with these inhibitors.

While our data suggest that HSV-1 assembly and release through the actin cortex in the neuronal cell body occurs independently of myosin II and V, we observed changes in the distribution of both myosin II and V in the cytoplasm of the cell body during HSV-1 infection. Both myosin II and myosin V were localized to the periphery of the cell body of mock-infected neurons; however, during HSV-1 infection, myosin II became more perinuclear in a striated pattern. This observation is in line with a report in which myosin II underwent redistribution from the cell periphery into a cage-like pattern during HSV-1 infection in epithelial cells [14]. Additionally, during HSV-1 infection, myosin V became concentrated in the nucleus where it associated with viral capsids. Similar observations have been reported in neurons infected with the closely related alphaherpesvirus, pseudorabies virus (PRV) [43] in which a strong association between nuclear actin filaments, myosin V and the PRV viral capsid protein VP26 was observed in the nucleus [43].

While myosin V may play a role and interact with HSV-1 capsids in the nucleus, our data suggests that this role may not be essential in sensory neurons as myosin V inhibition showed no significant effect on HSV-1 release from the nucleus or from cell body. These findings are consistent with previous observations that HSV-1 induces actin disassembly during virus egress which may facilitate virus exit [38]. Further work will be necessary to determine which, if any, HSV-1 capsid protein/s interact with myosin V in the nucleus of sensory neurons.

Our immunofluorescence studies, showing the presence of viral capsid and envelope protein pUS9 along axons, and TEM data, showing the presence of viral capsids in growth cones, in the presence or absence of actin and myosin inhibitors, indicate that anterograde transport of HSV-1 along axons to the growth cone occurs independently of actin, myosin II and myosin V. These findings complement our and other previous reports that anterograde transport along axons is largely mediated by microtubules and kinesins (such as kinesin-1 and -3) and reflects the dominance of microtubules as the main cytoskeletal component along the axon shaft [4–6,8,23,44–46]. In addition, our observations that the distribution of key cellular proteins involved in the secretory and exocytic pathways in neurons, SNAP-25, Rab6 and KIF3A along axons in the presence of actin and myosin V inhibitors was unaffected provides further evidence that their microtubule mediated transport was not affected by actin or myosin inhibition [33–35].

Growth cones are highly dynamic structures that facilitate axon growth and guidance and are the site of HSV-1 transmission from axons to epithelial cells [2]. Microtubules mainly terminate at the entrance to the growth cone, also known as the transition zone, and actin becomes the predominant cytoskeletal component [37]. Actin in the peripheral domain of the growth cone is arranged into an actin meshwork composed of branching fibres known as lamellipodia, and long F-actin bundles known as filopodia [37]. Very few microtubules extend into the peripheral domain along filopodia [37]. This suggests that HSV-1 must transition from microtubules to actin prior to final exocytosis. However, how HSV-1 is transported within the growth cone and the role of the actin cytoskeleton in the growth cone in virus exit have not been studied to date. An active treadmill of actin polymerization at the leading edge of the growth cone, and depolymerization at the entrance to the growth cone continuously remodels actin in the filopodia and lamellipodia resulting in an actin retrograde flow [47]. Our findings suggest that HSV-1 may utilize this actin treadmill for transport in the growth cone during virus exit.

The direct addition of actin polymerization inhibitors to axons disrupts actin filaments, halting the actin retrograde flow leading to growth cone and filopodia collapse [48,49]. Cytochalasin D and latrunculin A both inhibit actin polymerization and induce depolymerization by binding to F-actin and G-actin monomers, respectively [27,28]. Our results showed that inhibition of actin polymerization by either inhibitor resulted in a significant decrease in virus release from axons. Conversely, inhibition of actin branching using the actin related protein Arp2/3 complex inhibitor, CK-666, enhanced virus release. Arp2/3 inhibition increases the actin retrograde flow, resulting in retraction of the lamellipodia, as well as reduction of the lamellipodia density, without affecting F-actin in the filopodia [29,30]. This allows the entrance of microtubules beyond the transition zone and may increase HSV-1 access to long F-actin bundles, facilitating virus exit. Overall, our results suggest that that an intact actin cytoskeleton is required for virus release from growth cones and that HSV-1 utilizes this actin cytoskeleton for short-range transport in the growth cone.

Actin-based myosin molecular motors primarily mediate actin dynamics and cargo transport along actin filaments [50,51]. Our data showed that myosin V was essential for HSV-1 release from axonal varicosities and growth cones. Our immunofluorescence studies showed that myosin V colocalized with viral capsids in growth cones and this colocalization was prominent in myoVin1 treated axons. Myosin V is widely expressed in neurons and is abundant in the growth cones, facilitating cargo transport along F-actin in filopodia [10,17]. Myosin V is a processive motor with long lever arms enabling it to ‘walk’ across actin filaments, facilitating the transport of bulky cargo [52,53]. Moreover, myosin V is also involved in the delivery, tethering and docking of secretory vesicles to the cell surface during regulated exocytosis [54,55]. The recruitment of secretory vesicles to myosin V is mediated by RAB GTPases and Rab3A, Rab11 and Rab27 in post-Golgi secretory systems [56,57]. Myosin V also forms a multiprotein complex with vesicle-associated proteins VAMP/Synaptobrevin and Synaptophysin, which subsequently forms a SNARE complex with SNAP-25 [58–60]. We have previously shown that HSV-1 utilizes the large secretory vesicle pathway during anterograde axonal transport, with viral tegument and envelope proteins carried in vesicles associated with Rab3A, GAP-43, SNAP-25 and kinesin-1 [6]. Furthermore, a direct interaction between myosin Va and kinesin-1 in growth cones has been observed which may involve the switching of cargo from microtubules to actin [50]. This further supports the role that myosin V may play in the transport of HSV-1 within growth cones prior to final exocytosis. Further studies to determine how HSV-1 interacts with myosin V in growth cones are necessary to understand the mechanism of HSV-1 exit from axonal growth cones.

Our myosin II inhibition studies showed that myosin II does not play a role in HSV-1 egress from growth cones of sensory neurons. In growth cones, myosin II predominates in the transition zone and plays a key role in actin remodelling and dynamics. Myosin II is involved in actin arc formation, actin retrograde flow and actin bundle severing in the transition zone [11,51,61]. Additionally, myosin II coordinates actin and microtubules dynamics, restricting the entrance of microtubules into the growth cone [62]. Although myosin II does not appear to have a direct role in HSV-1 exit from growth cones, its inhibition can result in microtubules penetrating further into the growth cone periphery leading to increased length of actin bundles [32,61]. We observed a slight upward trend in virus release from growth cones following myosin II inhibition. It is possible that HSV-1 exit may be facilitated by longer actin bundles and by an increase in microtubules penetrating the periphery of the growth cones during inhibition of myosin II [11,30].

The findings presented here indicate that HSV-1 has adapted to utilize the unique actin cytoskeleton in the growth cone to facilitate its exit from axons and this can proceed without a requirement to contact epithelial cells. These findings are relevant to the process of virus release from nerves *in vivo* during recurrent herpes. Sensory nerve endings and epidermal keratinocytes do not appear to form classical synapses. However, they form close contact connections with membrane-to-membrane appositions [63–65]. Previous studies from our laboratory have shown that fully assembled viruses are released from axons into intercellular gaps before infecting epithelial cells *in vitro* [66]. Taken together, our studies have provided evidence for actin-based transport of HSV-1 within axonal growth cones of sensory neurons prior to final exocytosis, in addition to identifying myosin V as a key actin-associated molecular motor during this process. Overall, these findings contribute to the understanding of the mechanism(s) of HSV-1 exit and spread from nerve endings to epithelial cells during recurrent herpes.

## Supporting information

S1 Fig Schematic diagram showing the layout of the microfluidic neuronal devices. The neuronal device is divided into a cell body compartment (blue) and axonal compartment (yellow) These two compartments are connected by microgrooves, which allow the growth of axons from the cell body side to the axonal side. Shown are sensory DRG neurons at the time of seeding in the cell body compartment (left panel) and axon growth after 3 days in the axonal compartment (right panel). Scale bar = 100 μm.
(TIF)

S2 Fig Control assay to measure residual virus inoculum. Neonatal rat DRG neurons were dissociated and seeded into either the cell body compartment of microfluidic devices (40,000 cells), or on glass coverslips in a 24-well plate (20,000 cells per well). Neurons were infected with HSV-1 (1.7 x 10^6^ PFU in 350 μl for cultures in devices, or 0.85 x 10^6^ PFU in 500 μl for cultures on coverslips) and at 2 hours post-infection (hpi) the inoculum was collected. Neurons were washed twice with fresh media, followed by the addition of fresh media, which was collected at 2 hpi. Cultures were incubated until 30 hpi where the media was again collected. Viral DNA was extracted from the media and analysed by droplet digital PCR (ddPCR) to measure the copy number of DNA encoding for viral envelope protein, glycoprotein D (gD). Plotted are the averages of two samples ± the standard deviation for neuronal devices (circles) and coverslips (squares).
(TIF)

S3 Fig The myosin inhibitors, myoVin1 or *para*-aminoblebbistatin, do not affect viral replication or viral protein distribution in the cytoplasm of the cell body following treatment of axons. Neuronal cultures in the cell body compartment were HSV-1 or mock-infected and the axonal compartment was treated with either myoVin1 or *para*-aminoblebbistatin (pBlebb) at 6 hpi. Neuronal cultures were fixed at 30 hpi and immunostained for HSV-1 C capsids with either myosin V or myosin II. Cultures were examined using a Leica SP5 II confocal microscope. Micrographs of HSV-1 infected neurons showing label for viral envelope (green), viral capsid (red) and myosin V (cyan) following inhibition of myosin V by myoVin1, or myosin II (cyan) by *para-*aminoblebbistatin. Dashed lines outline the nucleus. Scale bars = 10 μm.
(TIF)

S4 Fig Actin and myosin inhibition does not affect the localization of KIF3A and SNAP25 along axons. Neuronal cultures in the cell body compartment were HSV-1 or mock-infected and both compartments were treated with either latrunculin A (Lat A, 2.5 μM) or myoVin1 (5 μM) at 6 hpi, or CK-666 (50 μM) at 22 hpi. Neuronal cultures were fixed at 30 hpi and immunostained for KIF3A and SNAP25. Cultures were examined using a Leica SP5 II confocal microscope. Micrographs of HSV-1 and mock-infected axons showing label for viral envelope (green), KIF3A (red) and SNAP25 (cyan) following inhibition of actin polymerization by latrunculin A, actin branching by CK-666, and of myosin V by myoVin1. Growth cones are indicated by arrows. Scale bars = 10 μm.
(TIF)

S5 Fig Actin and myosin inhibition does not affect the localization of Rab6 along axons. Neuronal cultures in the cell body compartment were HSV-1 or mock-infected and both compartments were treated with either latrunculin A (Lat A, 2.5 μM) or myoVin1 (5 μM) at 6 hpi or CK-666 (50 μM) at 22 hpi. Neuronal cultures were fixed at 30 hpi and immunostained for Rab6. Cultures were examined using a Leica SP5 II confocal microscope. Micrographs of HSV-1 and mock-infected axons showing label for viral envelope (green) and Rab6 (red) following inhibition of actin polymerization by latrunculin A, actin branching by CK-666, and of myosin V by myoVin1. Growth cones are indicated by arrows. Scale bars = 10 μm.
(TIF)
