# Supplementary figures and images for: Herpes simplex virus-1 utilizes the host actin cytoskeleton for its release from axonal growth cones
Source: PLoS Pathog. 2022 Jan 24;18(1):e1010264. doi: 10.1371/journal.ppat.1010264 (PMC8812851; doi:10.1371/journal.ppat.1010264)

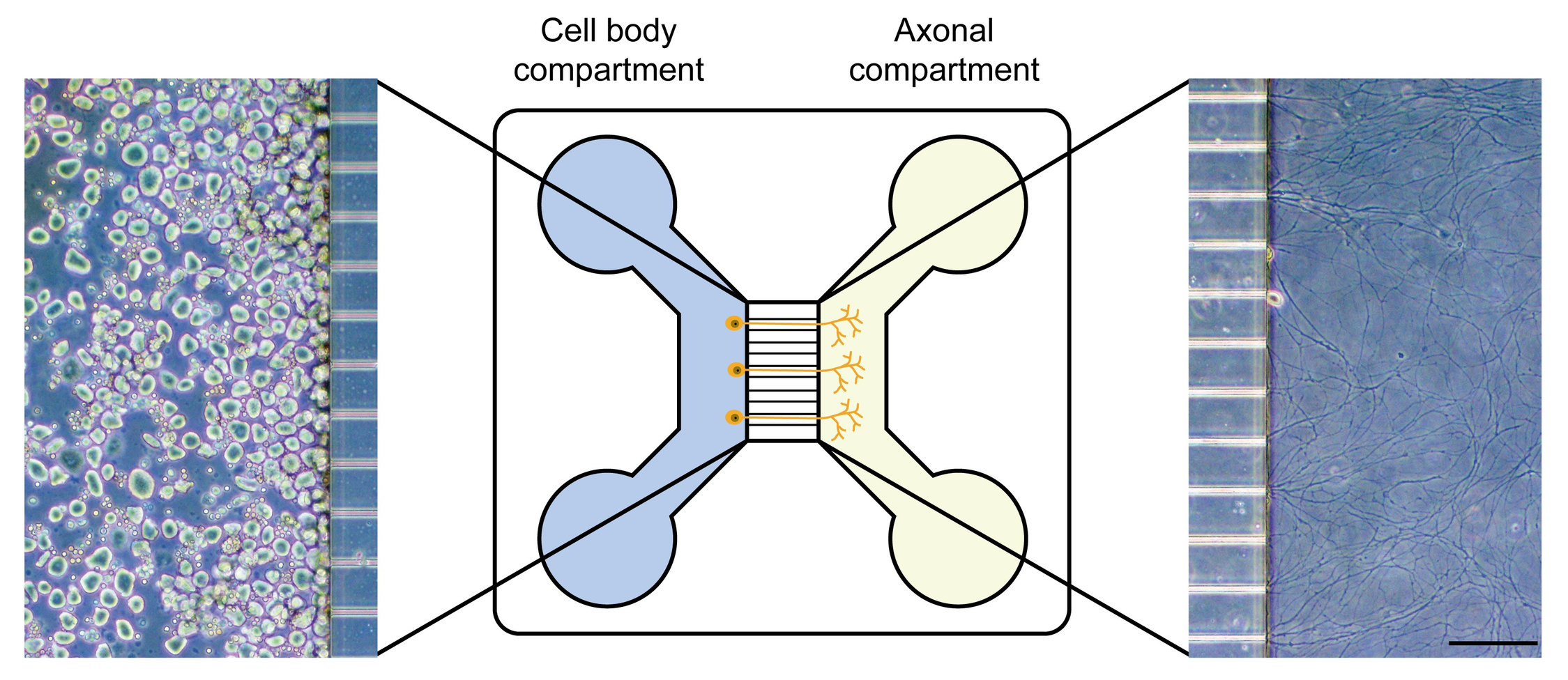

Supplement: S1 Fig — The neuronal device is divided into a cell body compartment (blue) and axonal compartment (yellow) These two compartments are connected by microgrooves, which allow the growth of axons from the cell body side to the axonal side. Shown are sensory DRG neurons at the time of seeding in the cell body compartment (left panel) and axon growth after 3 days in the axonal compartment (right panel). Scale bar = 100 μm. (TIF) [file ppat.1010264.s001.tif]

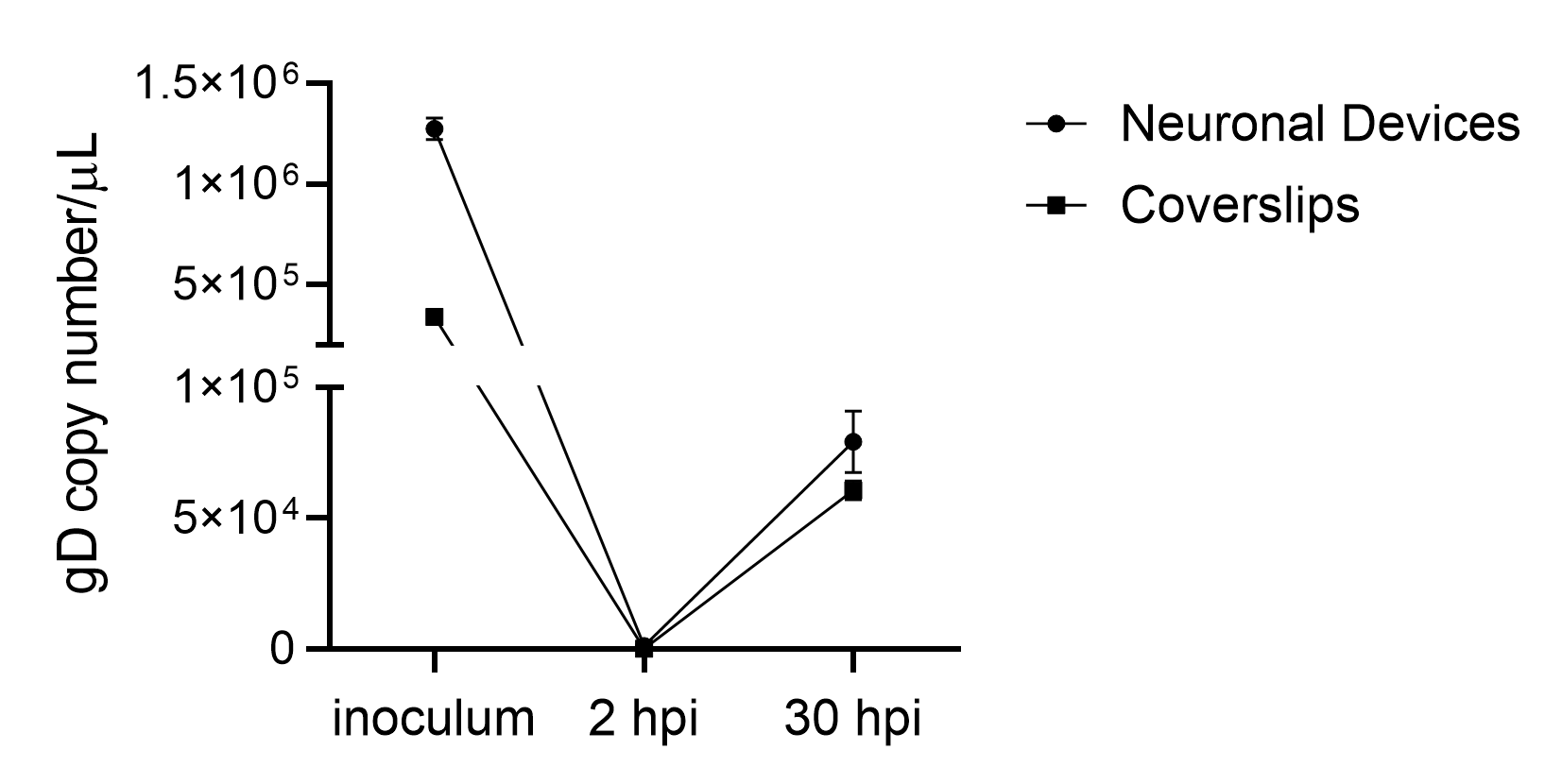

Supplement: S2 Fig — Neonatal rat DRG neurons were dissociated and seeded into either the cell body compartment of microfluidic devices (40,000 cells), or on glass coverslips in a 24-well plate (20,000 cells per well). Neurons were infected with HSV-1 (1.7 x 10^6^ PFU in 350 μl for cultures in devices, or 0.85 x 10^6^ PFU in 500 μl for cultures on coverslips) and at 2 hours post-infection (hpi) the inoculum was collected. Neurons were washed twice with fresh media, followed by the addition of fresh media, which was collected at 2 hpi. Cultures were incubated until 30 hpi where the media was again collected. Viral DNA was extracted from the media and analysed by droplet digital PCR (ddPCR) to measure the copy number of DNA encoding for viral envelope protein, glycoprotein D (gD). Plotted are the averages of two samples ± the standard deviation for neuronal devices (circles) and coverslips (squares). (TIF) [file ppat.1010264.s002.tif]

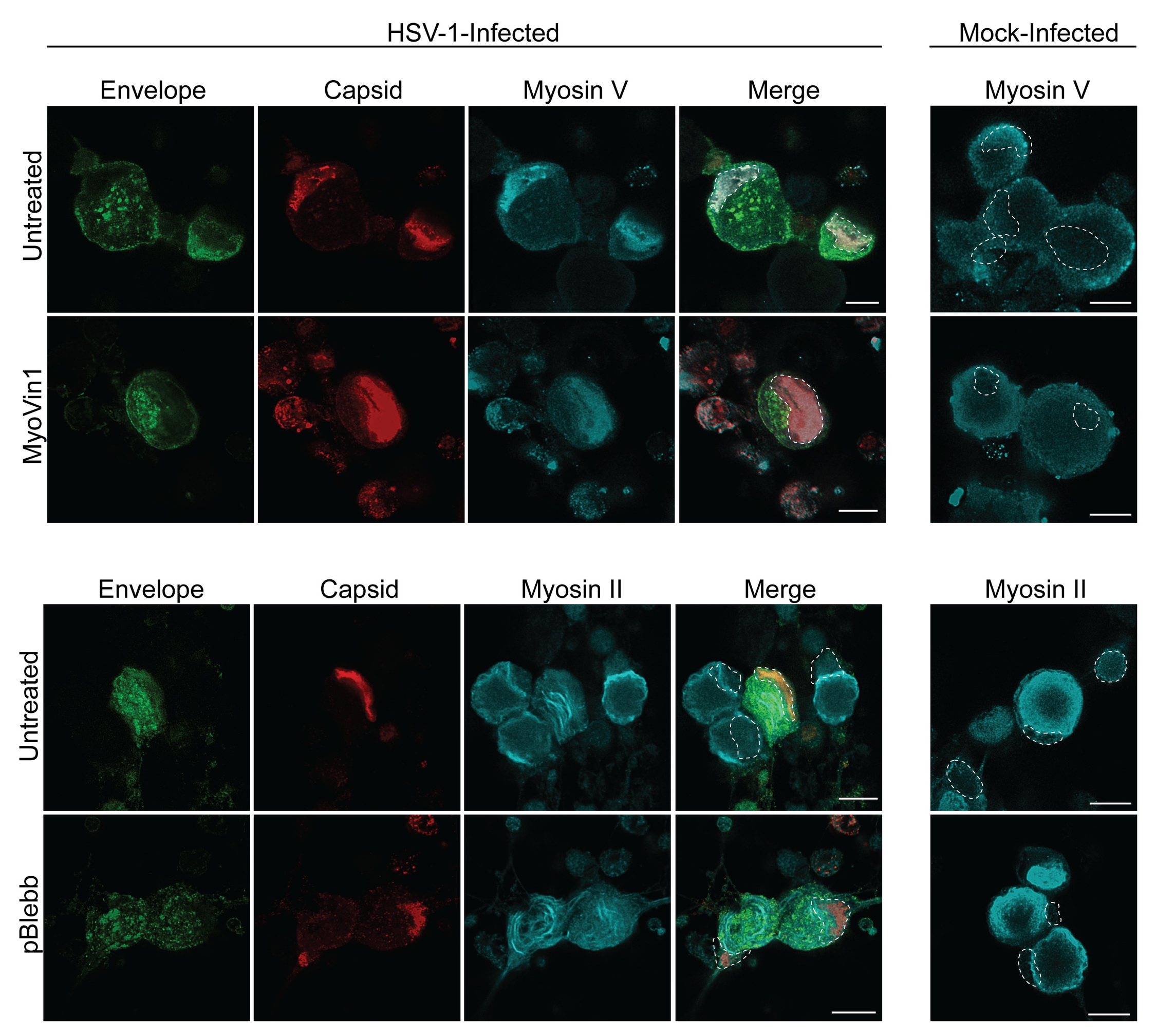

Supplement: S3 Fig — Neuronal cultures in the cell body compartment were HSV-1 or mock-infected and the axonal compartment was treated with either myoVin1 or para-aminoblebbistatin (pBlebb) at 6 hpi. Neuronal cultures were fixed at 30 hpi and immunostained for HSV-1 C capsids with either myosin V or myosin II. Cultures were examined using a Leica SP5 II confocal microscope. Micrographs of HSV-1 infected neurons showing label for viral envelope (green), viral capsid (red) and myosin V (cyan) following inhibition of myosin V by myoVin1, or myosin II (cyan) by para-aminoblebbistatin. Dashed lines outline the nucleus. Scale bars = 10 μm. (TIF) [file ppat.1010264.s003.tif]

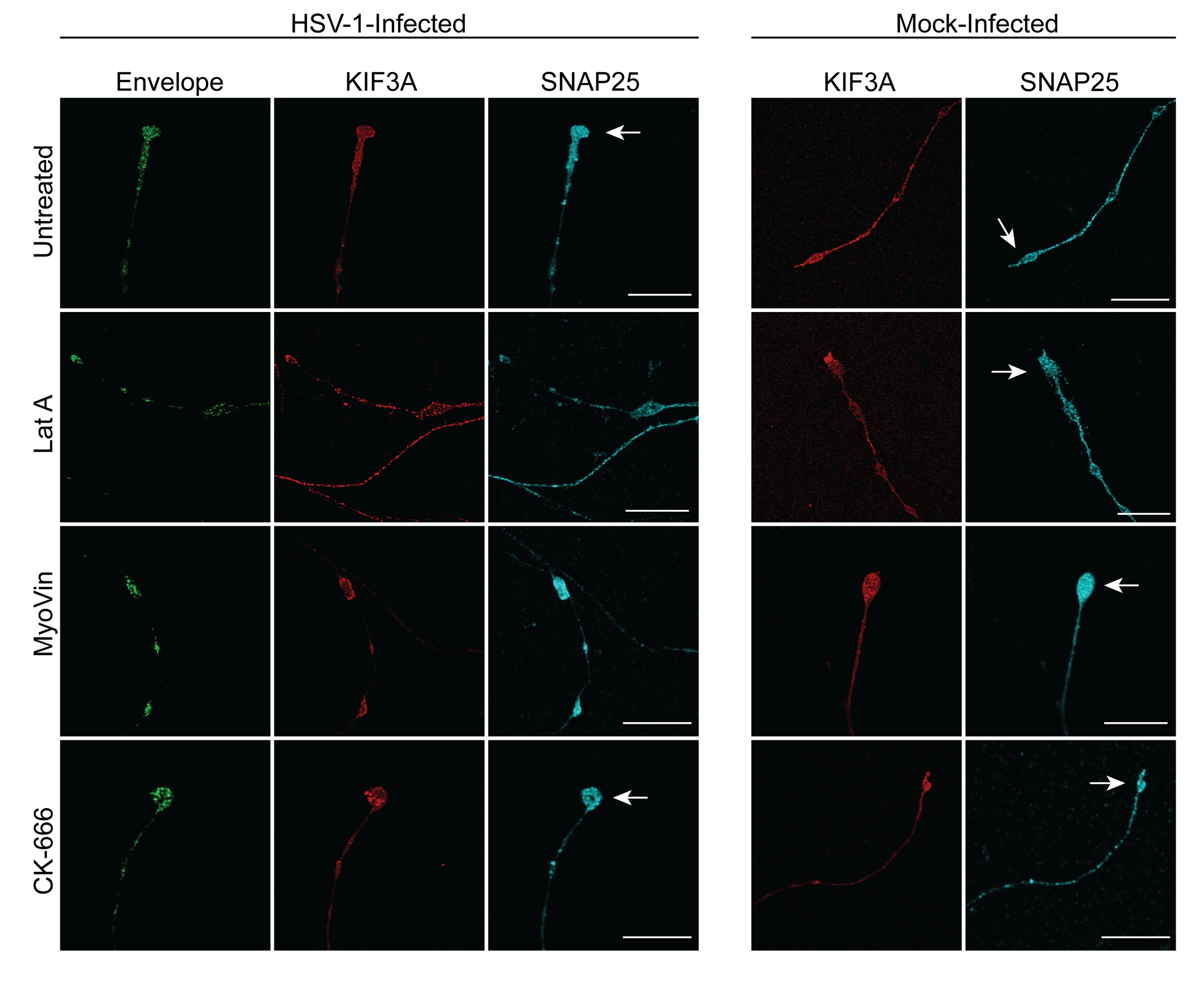

Supplement: S4 Fig — Neuronal cultures in the cell body compartment were HSV-1 or mock-infected and both compartments were treated with either latrunculin A (Lat A, 2.5 μM) or myoVin1 (5 μM) at 6 hpi, or CK-666 (50 μM) at 22 hpi. Neuronal cultures were fixed at 30 hpi and immunostained for KIF3A and SNAP25. Cultures were examined using a Leica SP5 II confocal microscope. Micrographs of HSV-1 and mock-infected axons showing label for viral envelope (green), KIF3A (red) and SNAP25 (cyan) following inhibition of actin polymerization by latrunculin A, actin branching by CK-666, and of myosin V by myoVin1. Growth cones are indicated by arrows. Scale bars = 10 μm. (TIF) [file ppat.1010264.s004.tif]

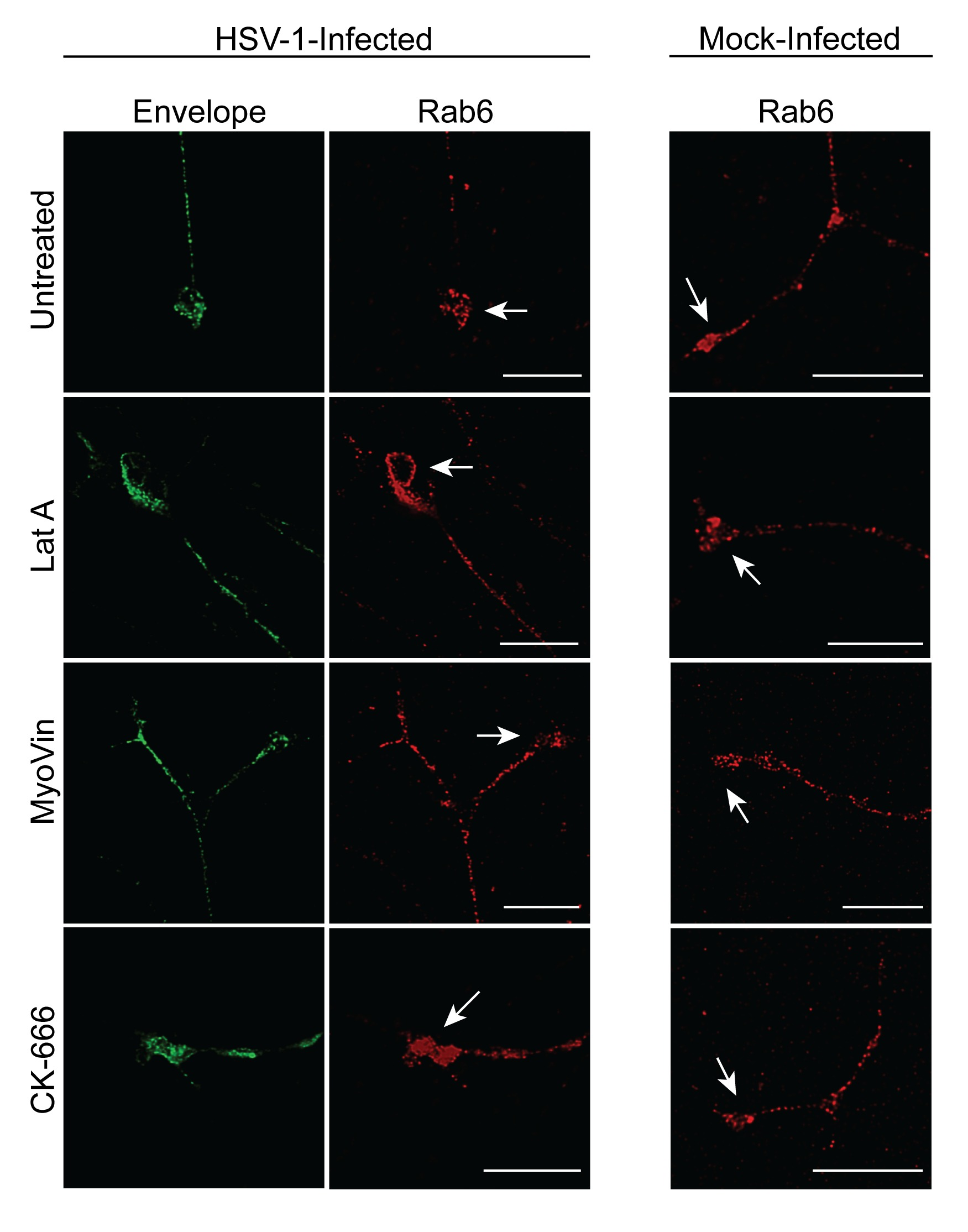

Supplement: S5 Fig — Neuronal cultures in the cell body compartment were HSV-1 or mock-infected and both compartments were treated with either latrunculin A (Lat A, 2.5 μM) or myoVin1 (5 μM) at 6 hpi or CK-666 (50 μM) at 22 hpi. Neuronal cultures were fixed at 30 hpi and immunostained for Rab6. Cultures were examined using a Leica SP5 II confocal microscope. Micrographs of HSV-1 and mock-infected axons showing label for viral envelope (green) and Rab6 (red) following inhibition of actin polymerization by latrunculin A, actin branching by CK-666, and of myosin V by myoVin1. Growth cones are indicated by arrows. Scale bars = 10 μm. (TIF) [file ppat.1010264.s005.tif]
